# Effect of screen-based simulation on pediatric drug administration among nursing students: a randomized controlled trial

**DOI:** 10.1590/1980-220X-REEUSP-2025-0249en

**Published:** 2025-12-01

**Authors:** Hilal Çelik Bayram, Sibel Ergün

**Affiliations:** 1 Balıkesir University, Faculty of Health Sciences, Department of Nursing, Division of Pediatric Nursing, Balıkesir, Türkiye.

**Keywords:** Pediatric Nursing, Education, Nursing, Drug Administration Routes, Pediatrics., Enfermagem Pediátrica, Educação em Enfermagem, Vias de Administração de Medicamentos, Pediatria.

## Abstract

**Objective:**

To evaluate the effect of screen-based simulation education on self-efficacy, student satisfaction, and self-confidence in learning related to pediatric drug administration among nursing students.

**Method:**

A randomized controlled design was used, involving an intervention group and a control group, to reach the target population of 206 nursing students. The study was registered at ClinicalTrials.gov (NCT06548659). Data were collected using the Sociodemographic Characteristics Form, the Student Satisfaction and Self Confidence in Learning Scale, and the Medication Administration Self Efficacy Scale in Children for Nursing Students.

**Results:**

In the intervention group, a statistically significant improvement was observed between pre-test and post-test scores. Post-test comparisons between the intervention and control groups revealed a significant increase in self-efficacy scores in the intervention group. Additionally, the intervention group demonstrated significantly higher post-test scores in student satisfaction and self-confidence in learning.

**Conclusion:**

Screen-based simulation education had a positive effect on nursing students’ self-efficacy in pediatric drug administration, as well as on satisfaction and confidence in the learning process.

## RESUMO

**Objetivo:**

Avaliar o efeito da simulação educacional baseada no ecrã na autoeficácia, satisfação dos alunos e autoconfiança na aprendizagem relacionada com a administração de medicação pediátrica entre estudantes de enfermagem.

**Método:**

Utilizou-se um desenho randomizado controlado, envolvendo um grupo de intervenção e um grupo de controlo, para atingir a população-alvo de 206 estudantes de enfermagem. O estudo foi registado no ClinicalTrials.gov (NCT06548659). Os dados foram recolhidos através do Formulário de Características Sociodemográficas, da Escala de Satisfação dos Alunos e Autoconfiança na Aprendizagem e da Escala de Autoeficácia na Administração de Medicamentos em Crianças para Estudantes de Enfermagem.

**Resultados:**

No grupo de intervenção observou-se uma melhoria estatisticamente significativa entre os scores pré e pós-teste. As comparações pós-teste entre os grupos intervenção e controlo revelaram um aumento significativo dos escores de autoeficácia no grupo intervenção. Além disso, o grupo de intervenção demonstrou pontuações pós-teste significativamente mais elevadas na satisfação dos alunos e na autoconfiança na aprendizagem.

**Conclusão:**

O ensino por simulação baseada em ecrã teve um efeito positivo na autoeficácia dos estudantes de enfermagem na administração de medicação pediátrica, bem como na satisfação e confiança no processo de aprendizagem.

## INTRODUCTION

Professional clinical practice opportunities are sometimes insufficient due to the high number of nursing students, limited availability of hospitals or clinical placement sites, shortages of qualified educators, and the shift to distance or blended learning models prompted by unexpected events such as the COVID-19 pandemic or natural disasters like earthquakes^(1,2,3)^. Thus, additional education techniques may be needed for both clinical and theoretical practices. Due to factors such as the suspension of face-to-face education, limited access to high-fidelity medical simulators, the high cost of simulation products, and a shortage of simulation-based learning specialists, nursing programs have increasingly adopted game-like, screen-based simulation tools. Examples commonly used in nursing education include vSim^®^ for Nursing, BodyInteract, and Full Code Medical Simulation^(4)^. Administering drugs to children differs from administering drugs to adults. Because pediatric patients constitute a more vulnerable population and often require complex and precise drug dose calculations, nursing students may experience heightened anxiety regarding medication administration in children. Therefore, it is essential that students receive effective training in pediatric drug administration, not only to ensure accuracy but also to foster student satisfaction. Integrating screen-based simulation technologies, which represent a contemporary and widely accepted educational approach, can play a vital role in enhancing both competence and confidence in this area^(5,6)^. Services provided in family health centers, newborn immunization programs, rabies vaccinations, flu vaccinations, home health care and drug therapy, the provision of insulin, the use of eye and ear drops, and school nursing are all examples of situations in which drugs may need to be administered to children^(7)^. Changes may be required in the educational materials used for nursing students to provide optimal patient care^(8)^. Computer- and screen-based simulations, which commonly incorporate a monitor, keyboard, virtual patients, fictional cases, virtual reality task trainers (training avatars), and staged virtual reality, offer an innovative approach to this process^(9)^. Various studies, such as video-based applications, have been conducted to enhance nursing students’ clinical outcomes and self-efficacy^(10)^. It is anticipated that screen-based simulation education will enhance nursing students’ self-efficacy and self-confidence in the learning process. In the gold standards published by the World Health Organization for nursing education, the importance of using simulation and e-learning techniques in nursing education models has been emphasized^(11)^. The literature indicates that nursing students are moderately, if not highly, satisfied with the verbal explanation, passive educational method. Low student satisfaction will negatively impact their learning confidence. Consequently, the quality of nursing education will suffer^(12)^. A study reported that traditional verbal instruction methods in nursing education were insufficient, whereas digital applications supported by various instructional models were found to enhance nursing students’ self-efficacy, clinical competencies, and non-technical skills^(13)^. It has also been determined that simulation-integrated postoperative patient care and advanced life support training contribute positively to students’ satisfaction and self-confidence in learning^(14,15,16)^. However, the number of studies conducted with simulations for the use of drugs in children is quite limited. Screen-based simulation, which can be utilized during extraordinary circumstances such as natural disasters and pandemics, has the potential to enhance nursing students’ self-efficacy and self-confidence in learning, particularly in situations where traditional education may be disrupted. The significance of screen-based simulation becomes even more pronounced under such conditions, representing a key strength of the present study. Notably, in Türkiye, pediatric medication administration has not previously been examined through the use of screen-based simulation tools. Furthermore, no existing studies have explored the effects of these technologies on nursing students’ self-efficacy, satisfaction, or self-confidence in learning related to pediatric drug administration. Accordingly, it was hypothesized that screen-based simulation education would have a positive effect on nursing students’ self-efficacy in pediatric drug administration, as well as on their satisfaction and self-confidence in learning. Therefore, this study aimed to evaluate the effect of screen-based simulation education on self-efficacy, student satisfaction, and self-confidence in learning related to pediatric drug administration among nursing students.

## METHOD

### Design of Study

This study employed a two-group, randomized controlled experimental design. The article was written and reported in accordance with the Simulation-Based Research Extensions of the CONSORT guideline^(17)^.

### Population

The study was conducted at the Faculty of Health Sciences at Balıkesir University, targeting a population of 206 pediatric nursing students. The aim was to include the entire population in the study. In the end, 198 second- and third-year nursing students who met the inclusion criteria and consented to participate were enrolled. Participants were randomly assigned to two groups: an intervention group and a control group, each comprising 99 students (n = 99 per group). Throughout the study, no participants withdrew or declined to continue.

### Blinding

Since the researcher managed the study process, researcher blinding could not be performed. A blind technique was used for the expert who performed the statistical analysis of the study and randomizing. The group to which the data belonged and the hypotheses of the study were kept confidential.

### Local

The study was conducted at the Faculty of Health Sciences of a state university in Türkiye between February 2024 and April 2024.

### Selection Criteria

The inclusion criteria for nursing students were as follows: being at least 18 years of age, providing informed consent to participate in the study, and having completed the theoretical course on pediatric drug administration delivered through verbal instruction, which involved oral explanations supported by slides and board presentations.

### Preparation of Screen-Based Simulation

The training simulation administered to the intervention group prior to data collection was developed by a nurse researcher with expertise in simulation design. The simulation scenarios were reviewed by a professor and an assistant professor from the Department of Child Health and Diseases, and the simulation was created with the assistance of a software development expert. The simulation was prepared in accordance with the good practice standards established by the International Nursing Association for Clinical Simulation and Learning^(18)^. During the design of the project, the training material was prepared by considering the UI (User Interface), UX (User Experience), color, and various other design techniques and rules. All of the components, photographs, and other products used in the simulation were taken from the database of the Canva Pro paid application and were originally designed by the researcher. The Lumi Education program was purchased and used for the software and interaction infrastructure, and the Voiser voice-over program was purchased and used for the voice-over of the avatar in the simulation. A total of four scenarios were created for the screen-based simulation. The descriptions of the topics covered in the scenarios were created by reviewing the literature, a drug administration guide, and textbooks^(19,20,21,22)^. Afterwards, expert opinion and approval regarding pediatric drug administration were obtained.

### Scenario Details

**Figure 1:**
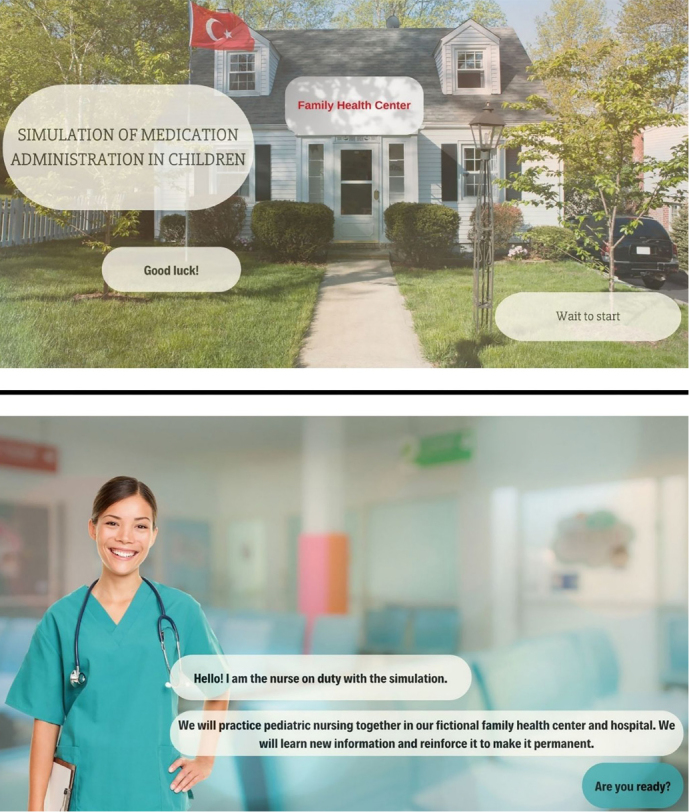
Visuals of the screen-based simulation.

***Scenario-1:*** This is the section where the avatar nurse introduces self and the simulation and discusses drug-drug interactions. The scenario takes place in a fictional Family Health Center. After teaching the interacting drugs, the student is expected to match the interacting drug groups (Figure 1).

***Scenario-2:*** This section takes place in a fictional Home Health Services Center. In this section, there are 8-month-old babies and 5-6-year-old children. This is the section where the nursing student is trained by the avatar on the safe doses of drugs for children and the calculation of the drug dose according to the child’s weight, and then the nursing students calculate the doses to be applied to the virtual patients to whom they will provide home health services.

**Figure 2:**
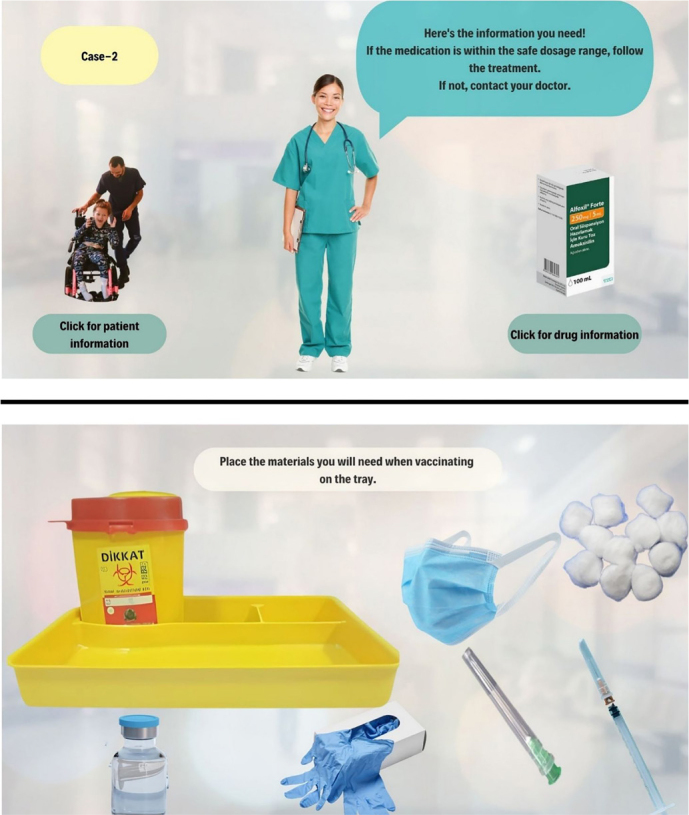
Visuals of the screen-based simulation.

***Senario-3:*** This is a section that takes place in a Home Health Services Center, including the Importance and Calculation of Dry Powder Volume in Children. In this section, there are child patients aged 2, 5, and 10. This is a section where the student calculates the drug dose for the treatment of patients whose homes she goes to provide home health services (Figure 2).

***Scenario-4:*** This is a section where nursing students learn the steps of drug administration in children. In this section, there are 2-month-old babies and children aged 5 and 14. This is the last section where the avatar nurse first provides information in the fictional Family Health Center and then expects the nursing student to list the steps.

### Data Collection Process Steps

**Step 1:** (Pre-test): Information about the study was provided to 206 nursing students Written signed consent was obtained from all students who agreed to participate in the study, and the Sociodemographic Characteristics Form, the Student Satisfaction and Self Confidence in Learning Scale (SCLS) and the Medication Administration Self-Efficacy Scale in Children for Nursing Students (MASSCNS) were pre-tested using the face-to-face interview technique and a questionnaire.

**Step 2:** The topic of pediatric drug administration was taught to all students in both the intervention and control groups through a four-hour classroom session using the verbal explanation method.

**Step 3:** Students who agreed to participate in the study and met the inclusion criteria were divided into the intervention and control groups using the online program at https://www. randomizer.org. Eight students who did not attend the course were excluded. Randomization was thus carried out on 198 individuals. The intervention group was trained through screen-based simulation on basic skills in pediatric drug administration, dose calculations, procedure steps, safe dose range calculation, preparation of materials for the procedure, etc., whereas the control group was not given simulation training at this stage of the study, and only received the verbal explanation method via lecture.

**Step 4:** Subgroups of ten people each from the 99-person intervention group were given screen-based simulation training in the computer laboratory located in the Faculty of Health Sciences building. A pilot study was conducted with the first subgroup. No negative feedback was received, and no problems were experienced in terms of comprehensibility and clarity. The data from the pilot study group were also included in the study. Groups participating in the computer laboratory were given 5 minutes of introductory information about the simulation, and then nursing students individually performed the pediatric medication administration simulation in the computer environment under the supervision of the researcher. The time required to complete each scenario varied by student, but was approximately 10–15 minutes. (The 4 scenarios lasted between 50 minutes and 1 hour in total.) After completing the four scenarios in the simulation, an information session was held in the form of a 5-minute group discussion on drug administration in children (basic skills in pediatric drug administration, dose calculations, procedure steps, calculation of the safe dose range, preparation of materials for the procedure). There were 10 nursing students in each session group and 9 in the last session.

**Figure 3:**
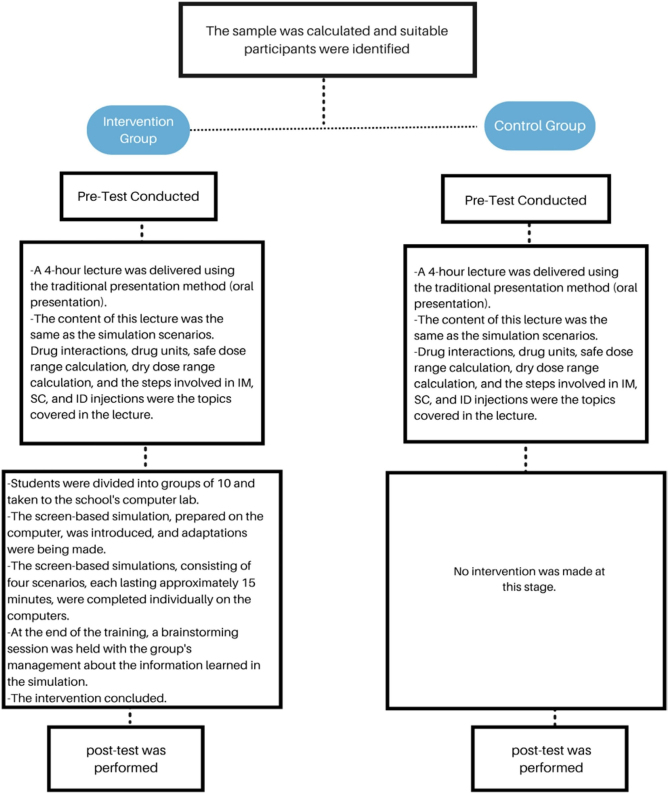
Flow chart summarizing pedagogical strategies.

**Step 5:** (Post-test): Following the simulation-based training, both groups were reassessed 20 days later using the SCLS and MASSCNS instruments through face-to-face interviews and surveys, and the results were subsequently analyzed. Upon completion of the data collection process, the control group received the pediatric drug administration training with the simulation, identical to the intervention group. No significant harm or adverse effects were observed in either group during the study. No significant harm or adverse effects were observed in either group during the study (Figure 3).

### Instruments

The data were collected using the “Sociodemographic Characteristics Form”, “Student Satisfaction and Self Confidence in Learning Scale (SCLS)”, “Medication Adminstration Self-Efficacy Scale in Children for Nursing Students (MASSCNS)”.

### Sociodemographic Characteristics Form

This form consisted of 16 questions about the demographic information of the students, including gender, age, marital status, high school graduation, and employment status^(23,24)^.

### Student Satisfaction and Self Confidence in Learning Scale (SCLS)

The scale was first published in 2014, and its Turkish version was tested for validity and reliability by Karaçay and Kaya in 2017. The scale has two sub-dimensions: satisfaction with learning and self-confidence. It is a five-point Likert-type scale consisting of 13 items, with the 13^th^ item being reverse-coded. The total possible score ranges from 13 to 65, with higher scores indicating greater satisfaction and self-confidence^(25)^. The internal consistency coefficient of the scale was reported as 0.94. In this study, the Cronbach’s alpha reliability coefficient was found to be 0.93 for both groups.

### Medication Administration Self-Efficacy Scale in Children For Nursing Students (Masscns)

Developed by Bektaş et al.^(26)^ in 2021, the scale comprises 16 items and includes two sub-dimensions: medication preparation for children and medication administration. It utilizes a five-point Likert-type scale, with a minimum possible score of 16 and a maximum of 80. The overall Cronbach’s alpha coefficient for the scale was reported as 0.94. In this study, the Cronbach’s alpha reliability coefficient was found to be 0.93 for the intervention group and 0.92 for the control group.

### Ethical Aspects

Before starting the study, ethical approval was obtained from the …… Ethics Committee (approval number E.272472, dated 11.07.2023), and institutional permission was granted by the …… Health Sciences Deanship (permission number E-23601865-100-295844, dated 22.09.2023). Participants were informed that their involvement was voluntary, that their personal information would be kept confidential, and that the data collected would be used solely for scientific research purposes. Written informed consent was obtained from all participants. Given the anticipated benefits of the simulation-based training, the control group also received the same simulation training upon completion of the study.

### Data Analysis and Treatment

All data were analyzed using SPSS software (version 22) on a Windows platform. Before analysis, assumptions for applying parametric or non-parametric tests were assessed. Normality of the data distribution was evaluated using the Kolmogorov-Smirnov test, complemented by an examination of skewness and kurtosis values. Paired-samples t-tests were conducted to examine within-group differences between pre-test and posttest scores, while independent-samples t-tests were employed to compare differences between groups. A significance level of 0.05 and a 95% confidence interval were set as the criteria for statistical significance.

## RESULTS

**Figure 4:**
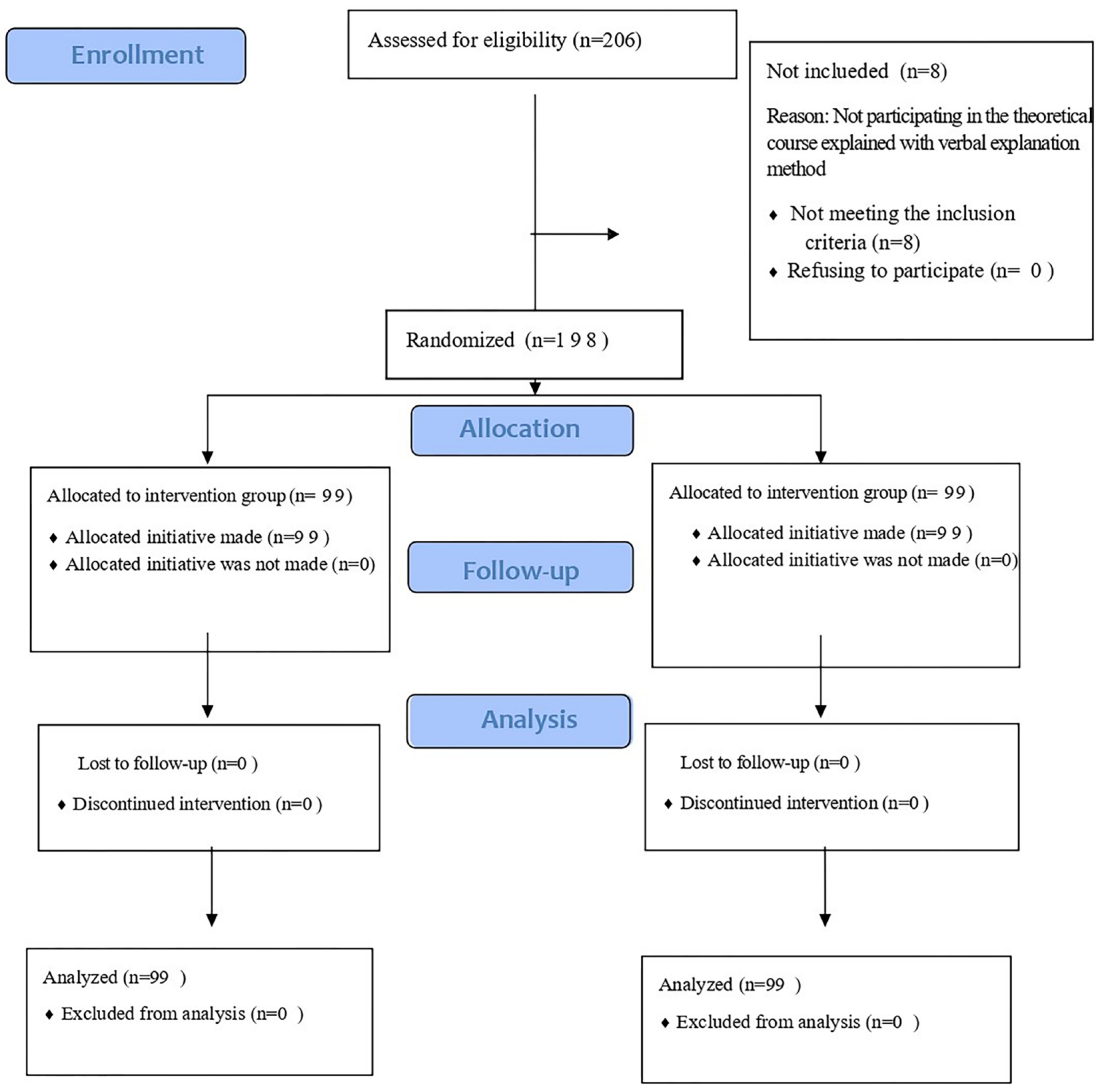
CONSORT flow diagram – *(NCT06548659) www.clinicaltrials.gov.tr*.

The study initially involved 206 second- and third-year nursing students. Eight students who did not meet the inclusion criteria were excluded. No participants refused to participate or withdrew from the study. Consequently, the study was completed with 99 nursing students in the intervention group and 99 in the control group (Figure 4).

Among the students, 52.70% (n: 78) of the female participants were in the intervention group, while 58% (n: 29) of the male participants were in the control group. Among the participants who were employed, 73.33% (n: 11) were in the intervention group, while 51.91% (n: 95) of the unemployed participants were in the control group. The mean age of the control group was 20.96 ± 1.19, and the mean age of the intervention group was 21.22 ± 2.34 (Table 1).

**Table 1:** Results regarding the comparison of demographic and categorical variables between the groups-Balıkesir – Marmara Region, Türkiye, 2023.

Variable		Group				t	p
		Control		Intervention			
		Mean ± SD		Mean ± SD			
Age		20.96 ± 1.19		21.22 ± 2.34		0.95	0.34
**Variable**	**Group**	**Groups**				**X^2^**	**p**
		**Control**		**Intervention**			
		**n**	**%**	**n**	**%**		
Gender	Female	70	47.30	78	52.70	1.71	0.19
	Male	29	58.00	21	42.00		
Marital status	Married	3	42.86	4	57.14	0.15	0.70
	Single	96	50.26	95	49.74		
High school type	Anatolian high school	88	52.07	81	47.93	2.79	0.25
	Health vocational high school	8	44.44	10	55.56		
	Science high school	3	27.27	8	72.73		
Employment status	Yes	4	26.67	11	73.33	3.53	0.06
	No	95	51.91	88	48.09		
Willingness to choose the nursing profession	Yes	46	52.27	42	47.73	0.33	0.85
	No	14	48.28	15	51.72		
	Neutral	39	48.15	42	51.85		
Frequency of engaging in simulations, educational games, etc., during nursing education.	Always	20	20.20	13	13.13	1.81	0.40
	Sometimes	66	60.67	71	71.72		
	Never	13	13.13	15	15.15		
Attitude toward nursing profession	Positive	48	48.98	50	51.02	1.85	0.40
	Negative	8	38.10	13	61.90		
	Neutral	43	54.43	36	45.57		

SD: Standard Deviation. X2: Chi square. t: Independent sample t test.

The mean post-test scores for the MASSCNS showed a statistically significant difference between the intervention and control groups (p < 0.05). The mean post-test score of the intervention group was found to be 68.08 ± 10.77. The mean posttest score for the same scale was 50.81 ± 13.91 in the control group, and the mean score of the intervention group was found to be statistically significantly higher. A statistically significant difference was found between the pre- and post-test scores for the MASSCNS in the intervention group (p < 0.05). When the mean values were examined, it was found that the mean pre-test scores of the intervention group (43.65 ± 14.08) were lower than the mean post-test scores (68.08 ± 10.77) (Table 2).

**Table 2:** Results regarding the comparison of the Self-Efficacy Scale for Medication Administration in Children for Nursing Students within the test time and between the groups-Balıkesir – Marmara Region, Türkiye, 2023.

	Group		Intergroup^**^
	Control	Intervention	
	Mean ± SD	Mean ± SD	
Student Satisfaction pre-test	16.81 ± 3.07	17.12 ± 3.51	t:–0.67; p:0.50
Student Satisfaction post-test	17.74 ± 3.21	23.88 ± 1.79	t:–16.64; **p:0.00**
Repeated Measures^*^	t:–2.32; p:0.02	t:–17.92; p:0.01	
Self-confidence in learning pre-test	27.14 ± 5.07	27.51 ± 5.29	t:–0.50; p:0.62
Self-confidence in learning post-test	27.79 ± 5.13	36.25 ± 3.73	t:–13.29; **p:0.00**
Repeated Measures^*^	t:–0.94; p:0.35	t:–14.53; p:0.01	
Student satisfaction and self-confidence in learning general pre-test	43.95 ± 7.73	44.35 ± 9.10	t:–0.34; p:0.74
Student satisfaction and self-confidence in learning general post-test	45.53 ± 7.79	60.13 ± 5.09	t:–15.61; **p:0.00**
Repeated Measures^*^	t:–1.54; p:0.13	t:–15.94; **p:0.01**	

SD: Standard Deviation.

^**^Independent Samples t-Test.

^*^Paired Samples t-Test p < 0.05.

The SCLS overall post-test mean scores showed a statistically significant difference between the groups (p < 0.05). It was determined that the SCLS overall post-test mean scores of the intervention group (60.13 ± 5.09) were higher than those of the control group (45.53 ± 7.79). A statistically significant difference was found between the SCLS general pre- and post-test mean scores of the intervention group (p < 0.05). When the mean values were examined, it was determined that the pre-test mean scores of the intervention group (44.35 ± 9.10) were lower than the post-test mean scores (60.13 ± 5.09) (Table 3).

**Table 3:** Results regarding the comparison of the Student Satisfaction and Self-Confidence in Learning Scale within time and between groups-Balıkesir – Marmara Region, Türkiye, 2023.

	Group		Intergroup^**^
	Control	Intervention	
	Mean ± SD	Mean ± SD	
Student Satisfaction pre-test	16.81 ± 3.07	17.12 ± 3.51	t:–0.67; p:0.50
Student Satisfaction post-test	17.74 ± 3.21	23.88 ± 1.79	t:–16.64; **p:0.00**
Repeated Measures^*^	t:–2.32; p:0.02	t:–17.92; p:0.01	
Self-confidence in learning pre-test	27.14 ± 5.07	27.51 ± 5.29	t:–0.50; p:0.62
Self-confidence in learning post-test	27.79 ± 5.13	36.25 ± 3.73	t:–13.29; **p:0.00**
Repeated Measures*	t:–0.94; p:0.35	t:–14.53; p:0.01	
Student satisfaction and self-confidence in learning general pre-test	43.95 ± 7.73	44.35 ± 9.10	t:–0.34; p:0.74
Student satisfaction and self-confidence in learning general post-test	45.53 ± 7.79	60.13 ± 5.09	t:–15.61; **p:0.00**
Repeated Measures^*^	t:–1.54; p:0.13	t:–15.94; **p:0.01**	

SD: Standard Deviation.

^**^Independent Samples t-Test.

^*^Paired Samples t-Test p < 0.05.

## DISCUSSION

This study investigated the effects of screen-based simulation education on self-efficacy and self-confidence in learning about pediatric drug administration among nursing students. When the MASSCNS findings were examined, the hypothesis “**H1_1_:** Screen-based simulation training has an effect on selfefficacy in pediatric drug administration in nursing students.” was confirmed.

While the self-efficacy of the control group, which received education via the verbal explanation method, showed an increase, the intervention group, educated through screen-based simulation, demonstrated a significantly greater improvement in self-efficacy. A difference in these scores has previously been associated with students being at the center of the learning process, having more freedom to make decisions, and the repeatable nature of the simulation^(27)^. It is thought that the interactive learning process involved in the simulation and the simulation having scenarios that encouraged clinical reasoning and critical thinking also contributed to the higher mean score of the intervention group^(27)^.

A study with similar characteristics to the present study and which was conducted to investigate the effect of a virtual game simulation on nursing diagnosis, goal setting, and diagnosis prioritization, found that the mean scores of the intervention group for nursing diagnosis and goal setting were significantly higher than those of the control group^(28)^. Although the education provided and the measurement and evaluation methods were different, the results of the study were similar to those of the present study.

The majority of studies conducted with different types of simulation on nursing students have evaluated the effect of simulations on self-efficacy, knowledge, and skills in different topics, and have revealed that the use of simulations made a significant difference^(14,28)^. Computer, web and screen-based simulations, which are different in name but have the same basic features, have mostly produced significantly high results in studies examining nursing self-efficacy, knowledge, and skills, as have other types of simulations^(29,30)^. Although procedures cannot be performed directly on a model as in high-fidelity simulations, the simplicity of this simulation method has been linked to meaningful outcomes, as it allows learners to work at their own pace in a safe environment and facilitates learning without risk of harm^(30)^.

It was determined that the satisfaction and self-confidence in learning of the intervention group students who received education with screen-based simulation were significantly higher. This result confirms the hypotheses “**H1_2_:** Education provided with screen-based simulation has an effect on student satisfaction” and “**H1_3_:** Education provided with screen-based simulation has an effect on self-confidence in learning in nursing students.”

The post-test score for the student satisfaction sub-dimension was higher in the intervention group than in the control group. This situation can be associated with the fact that the intervention group actively used the simulation, while the control group only received information about the simulation and did not receive effective training. The high student satisfaction in the intervention group can be related to the students’ general curiosity about educational technologies, their desire to have different experiences in education, the vibrant interface, which included a spoken voice application, and their sense of being at the center of the learning process^(28,30)^.

One study examining the effect of mobile application-based home visit training on students’ knowledge, competence, satisfaction, and self-confidence used an interactive video with a screen-based simulation feature^(31)^. It was found that this educational material increased the students’ satisfaction and self-confidence in learning. It was also effective in increasing the rate of learning, and there was a greater difference in the intervention group than in the control group, but this result was not significant. In line with these and other results, the result obtained in the present study is consistent with the increase in the average scores in the literature. An increase in student satisfaction and self-confidence in learning has been associated with students being able to pause videos, manage their own learning process, and use educational materials with colorful and eye-catching interfaces^(28,30)^. The higher average scores and significant differences in the present study can be associated with the simulation of the fictional Home Health Service and Family Health Center, the ability to repeat the process for each patient, and being able to use the educational material in a computer laboratory where there were no internet problems, rather than on a smartphone. An additional advantage of the present study was that the interactive elements were integrated throughout the simulation rather than being limited to the end. This approach helped maintain student engagement, minimizing distractions and contributing to a significant increase in both satisfaction and self-confidence within the learning subdimensions. Simulationbased education has become an important part of clinical education for various reasons, including the protection of patient rights and privacy, the lack of a sufficient number of teaching staff and practice areas, and the interruption of face-to-face education in extraordinary situations such as earthquakes and pandemics^(1,13)^. Screen-based simulations, which can be used by both individuals and groups, provide benefits because they can be accessed by the trainee via computer, tablet or phone at any time and place without face-to-face contact and are less expensive than high-fidelity simulators^(30,31)^.

### Limitations of the Study

The study was limited to students taking the pediatric nursing course at the Faculty of Health Sciences, University of Balıkesir. Therefore, the results can only be generalized to this study group. Another limitation of the study may be that the screen-based simulation does not fully reflect the real clinical environment.

## CONCLUSION

Nursing students report that children experience stress and low self-efficacy and self-confidence when administering medications due to factors such as children’s more sensitive nature and the difficulty of mathematical operations in medication preparation. This issue, as observed among nursing students, necessitates targeted intervention due to its potential impact on the quality of pediatric nursing care. Therefore, achieving high levels of self-efficacy, self-confidence, and satisfaction is essential for effective training in this area. Therefore, this study concluded that nursing students who received training in medication administration to children through screen-based simulation training had increased self-efficacy, student satisfaction, and learning confidence in administering medications to children compared to those who received training using verbal instruction.

In contexts where face-to-face education is not feasible, such as during disasters or pandemics, screen-based simulations offer accessible alternatives through widely used technological devices, including phones, tablets, and computers. This accessibility positively contributes to nursing education outcomes. The findings of this study are consistent with existing literature. Future research should explore screen-based simulations with nursing students not only on medication administration but also across diverse topics such as communication with pediatric patients and their families, therapeutic play, and the generalizability of these methods. Additionally, it is recommended that similar studies be conducted with larger sample sizes to enhance the generalizability of the findings.

## Data Availability

The datasets used in generating the findings of the study can be shared upon request from the authors.
