# Supplementary material for: Effect of screen-based simulation on pediatric drug administration among nursing students: a randomized controlled trial
Source: Rev Esc Enferm USP. 2025 Dec 1;59:e20250249. doi: 10.1590/1980-220X-REEUSP-2025-0249en (PMC12678002; doi:10.1590/1980-220X-REEUSP-2025-0249en)
Supplement: Translation

# Translation: Efecto de la simulación basada en pantalla sobre la administración de medicamentos pediátricos entre estudiantes de enfermería: ensayo controlado aleatorio

**DOI:** 10.1590/1980-220X-REEUSP-2025-0249es

**Authors:** Hilal Çelik Bayram, Sibel Ergün

**Affiliations:** 1 Balıkesir University, Faculty of Health Sciences, Department of Nursing, Division of Pediatric Nursing, Balıkesir, Türkiye.

**Keywords:** Enfermería Pediátrica, Educación en Enfermería, Vías de Administración de Medicamentos, Pediatría

## Abstract

**Objetivo:**

Evaluar el efecto de la educación mediante simulación en pantalla sobre la autoeficacia, la satisfacción estudiantil y la autoconfianza en el aprendizaje en relación con la administración de medicamentos pediátricos en estudiantes de enfermería.

**Métodos:**

Se utilizó un diseño controlado aleatorio, con un grupo de intervención y un grupo control, para llegar a la población objetivo de 206 estudiantes de enfermería. El estudio se registró en ClinicalTrials.gov (NCT06548659). Los datos se recopilaron mediante el Formulario de Características Sociodemográficas, la Escala de Satisfacción y Autoconfianza en el Aprendizaje del Estudiante y la Escala de Autoeficacia en la Administración de Medicamentos en Niños para Estudiantes de Enfermería.

**Resultados:**

En el grupo de intervención, se observó una mejora estadísticamente significativa entre las puntuaciones pretest y postest. Las comparaciones postest entre los grupos de intervención y control revelaron un aumento significativo en las puntuaciones de autoeficacia en el grupo de intervención. Además, el grupo de intervención mostró puntuaciones postest significativamente más altas en la satisfacción estudiantil y la autoconfianza en el aprendizaje.

**Conclusión:**

La educación con simulación basada en pantalla tuvo un efecto positivo en la autoeficacia de los estudiantes de enfermería en la administración de medicamentos pediátricos, así como en la satisfacción y la confianza en el proceso de aprendizaje.

## INTRODUCCIÓN

Las oportunidades de práctica clínica profesional son a veces insuficientes debido al elevado número de estudiantes de enfermería, la disponibilidad limitada de hospitales o centros de prácticas clínicas, la escasez de educadores cualificados y el cambio a modelos de aprendizaje a distancia o mixtos provocado por acontecimientos inesperados, como la pandemia de COVID-19 o desastres naturales como los terremotos^(1,2,3)^. Por lo tanto, es posible que se necesiten técnicas educativas adicionales tanto para las prácticas clínicas como para las teóricas. Debido a factores como la suspensión de la educación presencial, el acceso limitado a simuladores médicos de alta fidelidad, el alto costo de los productos de simulación y la escasez de especialistas en aprendizaje basado en la simulación, los programas de enfermería han adoptado cada vez más herramientas de simulación similares a los juegos y basadas en pantallas. Algunos ejemplos de uso común en la formación de enfermería son vSim^®^ for Nursing, BodyInteract y Full Code Medical Simulation^(4)^. La administración de medicamentos a niños difiere de la administración de medicamentos a adultos. Dado que los pacientes pediátricos constituyen una población más vulnerable y a menudo requieren cálculos complejos y precisos de la dosis de medicamentos, los estudiantes de enfermería pueden experimentar una mayor ansiedad en relación con la administración de medicamentos a niños. Por lo tanto, es esencial que los estudiantes reciban una formación eficaz en la administración de medicamentos pediátricos, no solo para garantizar la precisión, sino también para fomentar la satisfacción de los estudiantes. La integración de tecnologías de simulación por pantalla, que representan un enfoque educativo contemporáneo y ampliamente aceptado, puede desempeñar un papel fundamental en la mejora de la competencia y la confianza en este ámbito^(5,6)^. Los servicios prestados en los centros de salud familiar, los programas de inmunización de recién nacidos, las vacunas contra la rabia, las vacunas contra la gripe, la atención sanitaria a domicilio y la terapia farmacológica, el suministro de insulina, el uso de gotas para los ojos y los oídos y las enfermeras de programas escolares son ejemplos de situaciones en las que puede ser necesario administrar medicamentos a niños^(7)^. Es posible que sea necesario modificar los materiales educativos utilizados para los estudiantes de enfermería a fin de proporcionar una atención óptima a los pacientes^(8)^. Las simulaciones por computadora y en pantalla, que suelen incorporar la función de monitorear, un teclado, pacientes virtuales, casos ficticios, entrenadores de tareas de realidad virtual (avatares de entrenamiento) y realidad virtual escenificada, ofrecen un enfoque innovador para este proceso^(9)^. Se han realizado diversos estudios, como aplicaciones basadas en vídeos, para mejorar los resultados clínicos y la autoeficacia de los estudiantes de enfermería^(10)^. Se prevé que la educación mediante simulación basada en pantallas mejore la autoeficacia y la confianza de los estudiantes de enfermería en el proceso de aprendizaje. En las normas de referencia publicadas por la Organización Mundial de la Salud para la educación en enfermería, se ha destacado la importancia de utilizar técnicas de simulación y aprendizaje electrónico en los modelos de educación en enfermería^(11)^. La bibliografía indica que los estudiantes de enfermería están moderadamente, si no muy, satisfechos con la explicación verbal, un método educativo pasivo. La baja satisfacción de los estudiantes afectará negativamente a su confianza en el aprendizaje. En consecuencia, la calidad de la educación en enfermería se verá afectada^(12)^. Un estudio informó de que los métodos tradicionales de instrucción verbal en la educación en enfermería eran insuficientes, mientras que se descubrió que las aplicaciones digitales respaldadas por diversos modelos de instrucción mejoraban la autoeficacia, las competencias clínicas y las habilidades no técnicas de los estudiantes de enfermería^(13)^. También se ha determinado que la atención posoperatoria al paciente integrada en la simulación y la formación en soporte vital avanzado contribuyen positivamente a la satisfacción y la confianza de los estudiantes en el aprendizaje^(14,15,16)^. Sin embargo, el número de estudios realizados con simulaciones para el uso de medicamentos en niños es bastante limitado.

La simulación basada en pantallas, que puede utilizarse en circunstancias extraordinarias, como desastres naturales y pandemias, tiene el potencial de mejorar la autoeficacia y la confianza en el aprendizaje de los estudiantes de enfermería, especialmente en situaciones en las que la educación tradicional puede verse interrumpida. La importancia de la simulación basada en pantallas se hace aún más patente en tales condiciones, lo que representa una fortaleza clave del presente estudio. Cabe destacar que, en Turquía, la administración de medicamentos pediátricos no se ha examinado anteriormente mediante el uso de herramientas de simulación basadas en pantallas. Además, ningún estudio existente ha explorado los efectos de estas tecnologías en la autoeficacia, la satisfacción o la confianza en sí mismos de los estudiantes de enfermería en el aprendizaje relacionado con la administración de medicamentos pediátricos. En consecuencia, se planteó la hipótesis de que la educación mediante simulación en pantalla tendría un efecto positivo en la autoeficacia de los estudiantes de enfermería en la administración de medicamentos pediátricos, así como en su satisfacción y confianza en sí mismos en el aprendizaje. Por lo tanto, el objetivo de este estudio fue evaluar el efecto de la educación mediante simulación en pantalla sobre la autoeficacia, la satisfacción de los estudiantes y la confianza en sí mismos en el aprendizaje relacionado con la administración de medicamentos pediátricos entre los estudiantes de enfermería.

## MÉTODO

### Diseño Del Estudio

Este estudio empleó un diseño experimental controlado aleatorio de dos grupos. El artículo se redactó y presentó de acuerdo con las extensiones de investigación basadas en simulación de la guía CONSORT^(17)^.

### Población

El estudio se llevó a cabo en la Facultad de Ciencias de la Salud de la Universidad de Balıkesir, con una población objetivo de 206 estudiantes de enfermería pediátrica. El objetivo era incluir a toda la población en el estudio. Al final, se inscribieron 198 estudiantes de enfermería de segundo y tercer año que cumplían los criterios de inclusión y dieron su consentimiento para participar. Los participantes fueron asignados aleatoriamente a dos grupos: un grupo de intervención y un grupo de control, cada uno compuesto por 99 estudiantes (n = 99 por grupo). A lo largo del estudio, ningún participante se retiró ni se negó a continuar.

### Cegamiento

Dado que el investigador gestionó el proceso del estudio, no se pudo realizar el cegamiento del investigador. Se utilizó una técnica de cegamiento para el experto que realizó el análisis estadístico del estudio y la aleatorización. Se mantuvo la confidencialidad del grupo al que pertenecían los datos y las hipótesis del estudio.

### Local

El estudio se llevó a cabo en la Facultad de Ciencias de la Salud de una universidad estatal de Turquía entre febrero de 2024 y abril de 2024.

### Criterios de Selección

Los criterios de inclusión para los estudiantes de enfermería fueron los siguientes: tener al menos 18 años de edad, dar su consentimiento informado para participar en el estudio y haber completado el curso teórico sobre administración de medicamentos pediátricos impartido a través de instrucción verbal, que consistía en explicaciones orales apoyadas en diapositivas y presentaciones en pizarra.

### Preparación de la Simulación Basada en Pantalla

La simulación de formación administrada al grupo de intervención antes de la recolección de datos fue desarrollada por una enfermera investigadora con experiencia en el diseño de simulaciones. Los escenarios de simulación fueron revisados por un profesor y un profesor adjunto del Departamento de Salud y Enfermedades Infantiles, y la simulación se creó con la ayuda de un experto en desarrollo de software. La simulación se preparó de acuerdo con las normas de buenas prácticas establecidas por la Asociación Internacional de Enfermería para la Simulación Clínica y el Aprendizaje^(18^ Durante el diseño del proyecto, el material de formación se preparó teniendo en cuenta la interfaz de usuario (UI), la experiencia de usuario (UX), el color y otras técnicas y normas de diseño. Todos los componentes, fotografías y otros productos utilizados en la simulación se tomaron de la base de datos de la aplicación de pago Canva Pro y fueron diseñados originalmente por el investigador. Se adquirió y utilizó el programa Lumi Education para el software y la infraestructura de interacción, y se adquirió y utilizó el programa de voz en off Voiser para la voz en off del avatar en la simulación. Se crearon un total de cuatro escenarios para la simulación basada en pantalla. Las descripciones de los temas tratados en los escenarios se crearon revisando la bibliografía, una guía de administración de medicamentos y libros de texto^(19,20,21,22)^. Posteriormente, se obtuvo la opinión y la aprobación de expertos en administración de medicamentos pediátricos.

### Detalles del Escenario

***Escenario 1:*** En esta sección, la enfermera avatar se presenta a sí misma y a la simulación, y habla sobre las interacciones entre medicamentos. El escenario tiene lugar en un centro de salud familiar ficticio. Después de enseñar los medicamentos que interactúan, se espera que el estudiante empareje los grupos de medicamentos que interactúan (Figura 1).

**Figura 1:** Imágenes de la simulación en pantalla.

***Escenario 2:*** Esta sección tiene lugar en un centro ficticio de servicios de salud a domicilio. En esta sección hay bebés de 8 meses y niños de 5 a 6 años. En esta sección, el avatar forma al estudiante de enfermería sobre las dosis seguras de medicamentos para niños y el cálculo de la dosis del medicamento en función del peso del niño, y luego los estudiantes de enfermería calculan las dosis que se aplicarán a los pacientes virtuales a los que prestarán servicios de salud a domicilio.

***Escenario 3:*** Esta sección tiene lugar en un centro de servicios de salud a domicilio e incluye la importancia y el cálculo del volumen de polvo seco en niños. En esta sección hay pacientes infantiles de 2, 5 y 10 años. En esta sección, la estudiante calcula la dosis de medicamento para el tratamiento de los pacientes a cuyos hogares acude para prestarles servicios de salud a domicilio (Figura 2).

**Figura 2:** Imágenes de la simulación en pantalla.

***Escenario 4:*** En esta sección, los estudiantes de enfermería aprenden los pasos para administrar medicamentos a niños. En esta sección hay bebés de dos meses y niños de 5 y 14 años. Esta es la última sección en la que la enfermera avatar primero proporciona información en el centro de salud familiar ficticio y luego espera que el estudiante de enfermería enumere los pasos.

### Pasos Del Proceso Para Recolectar Datos

**Paso 1:** (Prueba previa): Se proporcionó información sobre el estudio a 206 estudiantes de enfermería. Se obtuvo el consentimiento por escrito de todos los estudiantes que aceptaron participar en el estudio, y se realizaron pruebas previas del Formulario de Características Sociodemográficas, la Escala de Satisfacción y Autoconfianza en el Aprendizaje (SCLS) y la Escala de Autoeficacia en la Administración de Medicamentos a Niños para Estudiantes de Enfermería (MASSCNS) mediante la técnica de entrevista cara a cara y un cuestionario.

**Paso 2:** Se impartió la materia de administración de medicamentos pediátricos a todos los estudiantes, tanto del grupo de intervención como del grupo de control, mediante una sesión presencial de cuatro horas utilizando el método de explicación verbal.

**Paso 3:** Los estudiantes que aceptaron participar en el estudio y cumplían los criterios de inclusión se dividieron en los grupos de intervención y control utilizando el programa en línea https://www.randomizer.org. Se excluyó a ocho estudiantes que no asistieron al curso. Así, se llevó a cabo la aleatorización de 198 personas. El grupo de intervención recibió formación mediante simulación en pantalla sobre habilidades básicas en la administración de medicamentos pediátricos, cálculo de dosis, pasos del procedimiento, cálculo del rango de dosis seguras, preparación de materiales para el procedimiento, etc., mientras que el grupo de control no recibió formación mediante simulación en esta fase del estudio, sino que solo recibió el método de explicación verbal a través de una conferencia.

**Paso 4:** Se impartió formación mediante simulación en pantalla a subgrupos de diez personas del grupo de intervención de 99 personas en el laboratorio de informática situado en el edificio de la Facultad de Ciencias de la Salud. Se realizó un estudio piloto con el primer subgrupo. No se recibieron comentarios negativos y no se experimentaron problemas en términos de comprensibilidad y claridad. Los datos del grupo del estudio piloto también se incluyeron en el estudio. Los grupos que participaron en el laboratorio de informática recibieron 5 minutos de información introductoria sobre la simulación y, a continuación, los estudiantes de enfermería realizaron individualmente la simulación de administración de medicamentos pediátricos en el entorno informático bajo la supervisión del investigador. El tiempo necesario para completar cada escenario varió según el estudiante, pero fue de aproximadamente 10-15 minutos. (Los 4 escenarios duraron entre 50 minutos y 1 hora en total). Después de completar los cuatro escenarios de la simulación, se celebró una sesión informativa en forma de debate grupal de 5 minutos sobre la administración de medicamentos en niños (habilidades básicas en la administración de medicamentos pediátricos, cálculos de dosis, pasos del procedimiento, cálculo del rango de dosis seguro, preparación de materiales para el procedimiento). Había 10 estudiantes de enfermería en cada grupo de la sesión y 9 en la última sesión.

**Paso 5:** (Prueba posterior): Tras la formación basada en la simulación, ambos grupos fueron reevaluados 20 días después utilizando los instrumentos SCLS y MASSCNS a través de entrevistas y encuestas presenciales, y posteriormente se analizaron los resultados. Una vez completado el proceso de recolección de datos, el grupo de control recibió la formación sobre la administración de medicamentos pediátricos con la simulación, idéntica a la del grupo de intervención. No se observaron daños significativos ni efectos adversos en ninguno de los dos grupos durante el estudio. No se observaron daños ni efectos adversos significativos en ninguno de los dos grupos durante el estudio (Figura 3).

**Figura 3:** Diagrama de flujo que resume las estrategias pedagógicas.

### Instrumentos

Los datos se recolectaron utilizando el “Formulario de características sociodemográficas”, la “Escala de satisfacción y confianza en sí mismo en el aprendizaje (Student Satisfaction and Self Confidence in Learning Scale - SCLS)” y la “Escala de autoeficacia en la administración de medicamentos a niños para estudiantes de enfermería (Medication Administration SelfEfficacy Scale in Children for Nursing Students - MASSCNS)”.

### Formulario de Características Sociodemográficas

Este formulario constaba de 16 preguntas sobre la información demográfica de los estudiantes, incluyendo sexo, edad, estado civil, graduación de secundaria y situación laboral^(23,24)^.

### Escala de Satisfacción y Confianza en el Aprendizaje de los Estudiantes (Scls)

La escala se publicó por primera vez en 2014, y Karaçay y Kaya comprobaron la validez y fiabilidad de su versión turca en 2017. La escala tiene dos subdimensiones: satisfacción con el aprendizaje y confianza en sí mismo. Se trata de una escala tipo Likert de cinco puntos que consta de 13 ítems, siendo el 13.º ítem de codificación inversa. La puntuación total posible oscila entre 13 y 65, y las puntuaciones más altas indican una mayor satisfacción y confianza en sí mismo^(25)^. El coeficiente de consistencia interna de la escala fue de 0,94. En este estudio, seencontró que el coeficiente de fiabilidad alfa de Cronbach era de 0,93 para ambos grupos.

### Escala de Autoeficacia en la Administración de Medicamentos a Niños Para Estudiantes de Enfermería (Masscns)

Desarrollada por Bektaş et al.^(26)^ en 2021, la escala consta de 16 ítems e incluye dos subdimensiones: preparación de medicamentos para niños y administración de medicamentos. Utiliza una escala tipo Likert de cinco puntos, con una puntuación mínima posible de 16 y una máxima de 80. El coeficiente alfa de Cronbach global de la escala fue de 0,94. En este estudio, el coeficiente de fiabilidad alfa de Cronbach fue de 0,93 para el grupo de intervención y de 0,92 para el grupo de control.

### Aspectos Éticos

Antes de iniciar el estudio, se obtuvo la aprobación ética del Comité de Ética (número de aprobación E.272472, con fecha del 11.07.2023) y se concedió el permiso institucional por parte de la Decanatura de Ciencias de la Salud (número de permiso E-23601865-100-295844, con fecha del 22.09.2023). Se informó a los participantes de que su participación era voluntaria, que su información personal se mantendría confidencial y que los datos para recolectar se utilizarían únicamente con fines de investigación científica. Se obtuvo el consentimiento informado por escrito de todos los participantes. Dados los beneficios previstos de la formación basada en simulaciones, el grupo de control también recibió la misma formación tras la finalización del estudio.

### Análisis y Tratamiento de Los Datos

Todos los datos se analizaron utilizando el software SPSS (versión 22) en una plataforma Windows. Antes del análisis, se evaluaron los supuestos para aplicar pruebas paramétricas o no paramétricas. La normalidad de la distribución de los datos se evaluó mediante la prueba de Kolmogorov-Smirnov, complementada con un examen de los valores de asimetría y curtosis. Se realizaron pruebas t para muestras emparejadas para examinar las diferencias dentro del grupo entre las puntuaciones previas y posteriores a la prueba, mientras que se emplearon pruebas t para muestras independientes para comparar las diferencias entre los grupos. Se estableció un nivel de significación de 0,05 y un intervalo de confianza del 95% como criterios de significación estadística.

## RESULTADOS

En el estudio participaron inicialmente 206 estudiantes de enfermería de segundo y tercer año. Se excluyó a ocho estudiantes que no cumplían los criterios de inclusión. Ningún participante se negó a participar ni se retiró del estudio. En consecuencia, el estudio se completó con 99 estudiantes de enfermería en el grupo de intervención y 99 en el grupo de control (Figura 4).

**Figura 4:** Diagrama de flujo Consort –(NCT06548659) www.clinicaltrials.gov.tr.

Entre los estudiantes, el 52,70% (n: 78) de las participantes femeninas formaban parte del grupo de intervención, mientras que el 58% (n: 29) de los participantes masculinos formaban parte del grupo de control. Entre los participantes que tenían empleo, el 73,33% (n: 11) formaba parte del grupo de intervención, mientras que el 51,91% (n: 95) de los participantes desempleados formaba parte del grupo de control. La edad media del grupo de control era de 20,96 ± 1,19, y la edad media del grupo de intervención era de 21,22 ± 2,34 (Tabla 1).

**Tabla 1:** Resultados de la comparación de variables demográficas y categóricas entre los grupos: Balıkesir - Marmara Bölgesi, Turquía, 2023.

Variable		Grupo				t	p
		Control		Intervención			
		Media ± DE		Media ± DE			
Edad		20.96 ± 1.19		21.22 ± 2.34		0.95	0.34
**Variable**	**Grupo**	**Grupos**				**X^2^**	**p**
		**Control**		**Intervención**			
		**n**	**%**	**n**	**%**		
Género	Femenino	70	47.30	78	52.70	1.71	0.19
	Masculino	29	58.00	21	42.00		
Estado civil	Casado	3	42.86	4	57.14	0.15	0.70
	Soltero	96	50.26	95	49.74		
Tipo de escuela secundaria	Escuela secundaria de Anatolia	88	52.07	81	47.93	2.79	0.25
	Secundaria Vocacional de salud	8	44.44	10	55.56		
	Secundaria científica	3	27.27	8	72.73		
Empleo	Si	4	26.67	11	73.33	3.53	0.06
	No	95	51.91	88	48.09		
Interés en la profesión de enfermería	Si	46	52.27	42	47.73	0.33	0.85
	No	14	48.28	15	51.72		
	Neutral	39	48.15	42	51.85		
Frecuencia con la que se realizan simulaciones, juegos educativos, etc., durante la formación en enfermería.	Siempre	20	20.20	13	13.13	1.81	0.40
	A veces	66	60.67	71	71.72		
	Nunca	13	13.13	15	15.15		
Actitud hacia la profesión de enfermería	Positiva	48	48.98	50	51.02	1.85	0.40
	Negativa	8	38.10	13	61.90		
	Neutral	43	54.43	36	45.57		

DE: Desviación estándar. X2: Chi cuadrado.

Las puntuaciones medias posteriores a la prueba del MASSCNS mostraron una diferencia estadísticamente significativa entre los grupos de intervención y control (p < 0,05). La puntuación media posterior a la prueba del grupo de intervención fue de 68,08 ± 10,77. La puntuación media posterior a la prueba para la misma escala fue de 50,81 ± 13,91 en el grupo de control, y se observó que la puntuación media del grupo de intervención era estadísticamente significativa más alta. Se observó una diferencia estadísticamente significativa entre las puntuaciones previas y posteriores a la prueba para el MASSCNS en el grupo de intervención (p < 0,05). Al examinar los valores medios, se observó que las puntuaciones medias previas a la prueba del grupo de intervención (43,65 ± 14,08) eran inferiores a las puntuaciones medias posteriores a la prueba (68,08 ± 10,77) (Tabla 2).

**Tabla 2:** Resultados de la comparación de la Escala de Autoeficacia para la Administración de Medicamentos en Niños para Estudiantes de Enfermería dentro del tiempo de prueba y entre los grupos: Bal**i**kesir - Marmara Bolgesi, Turquía, 2023.

	Grupo		Intergrupos^**^
	Control	Intervención	
	Media ± DE	Media ± DE	
Satisfacción del estudiante pre-test	16.81 ± 3.07	17.12 ± 3.51	t:–0.67; p:0.50
Satisfacción del estudiante post-test	16.81 ± 3.07	17.12 ± 3.51	t:–0.67; p:0.50
Mediciones repetidas^*^	t:–2.32; p:0.02	t:–17.92; p:0.01	
Autoconfianza en aprendizaje post-test	27.14 ± 5.07	27.51 ± 5.29	t:–0.50; p:0.62
Autoconfianza en aprendizaje post-test	27.14 ± 5.07	27.51 ± 5.29	t:–0.50; p:0.62
Mediciones repetidas^*^	t:–0.94; p:0.35	t:–14.53; p:0.01	
Satisfacción del estudiante y Autoconfianza en aprendizaje general pre-test	43.95 ± 7.73	44.35 ± 9.10	t:–0.34; p:0.74
Satisfacción del estudiante y Autoconfianza en aprendizaje general post-test	45.53 ± 7.79	60.13 ± 5.09	t:–15.61; **p:0.00**
Mediciones repetidas^*^	t:–1.54; p:0.13	t:–15.94; **p:0.01**	

DE: Desviación estándar.

^**^Prueba t para muestras independientes.

^*^Prueba t para muestras pareadas p < 0,05.

Las puntuaciones medias generales de la prueba posterior al SCLS mostraron una diferencia estadísticamente significativa entre los grupos (p < 0,05). Se determinó que las puntuaciones medias generales de la prueba posterior al SCLS del grupo de intervención (60,13 ± 5,09) eran más altas que las del grupo de control (45,53 ± 7,79). Se encontró una diferencia estadísticamente significativa entre las puntuaciones medias generales previas y posteriores a la prueba SCLS del grupo de intervención (p < 0,05). Al examinar los valores medios, se determinó que las puntuaciones medias previas a la prueba del grupo de intervención (44,35 ± 9,10) eran inferiores a las puntuaciones medias posteriores a la prueba (60,13 ± 5,09) (Tabla 3).

**Tabla 3:** Resultados de la comparación de la Escala de Satisfacción y Autoconfianza en el Aprendizaje de los Estudiantes a lo largo del tiempo y entre grupos: Bal**i**kesir - Marmara Bolgesi, Turquía, 2023.

	Grupo		Intergrupos^**^
	Control	Intervención	
	Media ± DE	Media ± DE	
Satisfacción del estudiante pre-test	16.81 ± 3.07	17.12 ± 3.51	t:–0.67; p:0.50
Satisfacción del estudiante post-test	17.74 ± 3.21	23.88 ± 1.79	t:–16.64; **p:0.00**
Mediciones repetidas^*^	t:–2.32; p:0.02	t:–17.92; p:0.01	
Autoconfianza en aprendizaje pre-test	27.14 ± 5.07	27.51 ± 5.29	t:–0.50; p:0.62
Autoconfianza en aprendizaje post-test	27.79 ± 5.13	36.25 ± 3.73	t:–13.29; **p:0.00**
Mediciones repetidas*	t:–0.94; p:0.35	t:–14.53; p:0.01	
Satisfacción del estudiante y Autoconfianza en aprendizaje general pre-test	43.95 ± 7.73	44.35 ± 9.10	t:–0.34; p:0.74
Satisfacción del estudiante y Autoconfianza en aprendizaje general post-test	45.53 ± 7.79	60.13 ± 5.09	t:–15.61; **p:0.00**
Mediciones repetidas^*^	t:–1.54; p:0.13	t:–15.94; **p:0.01**	

DE: Desviación estándar.

^**^Prueba t para muestras independientes.

^*^Prueba t para muestras pareadas p < 0,05.

## DISCUSIÓN

Este estudio investigó los efectos de la educación mediante simulación en pantalla sobre la autoeficacia y la confianza en sí mismos de los estudiantes de enfermería en el aprendizaje de la administración de medicamentos pediátricos. Al examinar los resultados del MASSCNS, se confirmó la hipótesis **“H1i:**La formación mediante simulación en pantalla tiene un efecto sobre la autoeficacia en la administración de medicamentos pediátricos en los estudiantes de enfermería”.

Mientras que la autoeficacia del grupo de control, que recibió formación mediante explicaciones verbales, mostró un aumento, el grupo de intervención, formado mediante simulación basada en pantalla, demostró una mejora significativamente mayor en la autoeficacia. Anteriormente, se había asociado la diferencia en estas puntuaciones con el hecho de que los estudiantes fueran el centro del proceso de aprendizaje, tuvieran más libertad para tomar decisiones y la naturaleza repetible de la simulación^(27)^. Se cree que el proceso de aprendizaje interactivo que implica la simulación y el hecho de que esta contuviera escenarios que fomentaban el razonamiento clínico y el pensamiento crítico también contribuyeron a la puntuación media más alta del grupo de intervención^(27)^.

Un estudio con características similares al presente y que se llevó a cabo para investigar el efecto de una simulación de juego virtual en el diagnóstico de enfermería, el establecimiento de objetivos y la priorización de diagnósticos, encontró que las puntuaciones medias del grupo de intervención para el diagnóstico de enfermería y el establecimiento de objetivos eran significativamente más altas que las del grupo de control^(28)^. Aunque la educación impartida y los métodos de medición y evaluación eran diferentes, los resultados del estudio fueron similares a los del presente.

La mayoría de los estudios realizados con diferentes tipos de simulación en estudiantes de enfermería han evaluado el efecto de las simulaciones en la autoeficacia, los conocimientos y las habilidades en diferentes temas, y han revelado que el uso de simulaciones marcó una diferencia significativa^(14,28)^. Las simulaciones por computadora, web y pantalla, que son diferentes en nombre pero tienen las mismas características básicas, han producido en su mayoría resultados significativamente altos en los estudios que examinan la autoeficacia, los conocimientos y las habilidades de enfermería, al igual que otros tipos de simu- laciones^(29,30)^. Aunque los procedimientos no pueden realizarse directamente en un modelo como en las simulaciones de alta fidelidad, la simplicidad de este método de simulación se ha relacionado con resultados significativos, ya que permite a los alumnos trabajar a su propio ritmo en un entorno seguro y facilita el aprendizaje sin riesgo de daños^(30)^.

Se determinó que la satisfacción y la confianza en sí mismos en el aprendizaje de los estudiantes del grupo de intervención que recibieron educación con simulación basada en pantalla eran significativamente mayores. Este resultado confirma las hipótesis “**H1_2_:** La educación impartida con simulación basada en pantalla tiene un efecto en la satisfacción de los estudiantes” y “**H1_3_:** La educación impartida con simulación basada en pantalla tiene un efecto en la confianza en el aprendizaje de los estudiantes de enfermería”.

La puntuación de la prueba posterior para la subdimensión de satisfacción de los estudiantes fue mayor en el grupo de intervención que en el grupo de control. Esta situación puede estar relacionada con el hecho de que el grupo de intervención utilizó activamente la simulación, mientras que el grupo de control solo recibió información sobre la simulación y no recibió una formación eficaz. La alta satisfacción de los estudiantes del grupo de intervención puede estar relacionada con la curiosidad general de los estudiantes por las tecnologías educativas, su deseo de tener experiencias diferentes en la educación, la interfaz dinámica, que incluía una aplicación de voz hablada, y su sensación de estar en el centro del proceso de aprendizaje^(28,30)^.

Un estudio que examinó el efecto de la formación sobre visitas domiciliarias basada en aplicaciones móviles en los conocimientos, la competencia, la satisfacción y la confianza en sí mismos de los estudiantes utilizó un video interactivo con una función de simulación en pantalla^(30)^. Se descubrió que este material educativo aumentaba la satisfacción y la confianza en sí mismos de los estudiantes en el aprendizaje. También fue eficaz para aumentar la tasa de aprendizaje, y hubo una mayor diferencia en el grupo de intervención que en el grupo de control, pero este resultado no fue significativo. En consonancia con estos y otros resultados, el resultado obtenido en el presente estudio es coherente con el aumento de las puntuaciones medias en la bibliografía. El aumento de la satisfacción y la confianza en sí mismos de los estudiantes en el aprendizaje se ha asociado con la posibilidad de pausar los videos, gestionar su propio proceso de aprendizaje y utilizar materiales educativos con interfaces coloridas y llamativas^(28,30)^. Las puntuaciones medias más altas y las diferencias significativas del presente estudio pueden asociarse con la simulación del Servicio de Salud Domiciliaria y el Centro de Salud Familiar ficticios, la posibilidad de repetir el proceso para cada paciente y la posibilidad de utilizar el material educativo en un laboratorio de informática donde no había problemas de conexión a Internet, en lugar de en un teléfono inteligente. Una ventaja adicional del presente estudio fue que los elementos interactivos se integraron a lo largo de toda la simulación, en lugar de limitarse al final. Este enfoque ayudó a mantener el interés de los estudiantes, minimizando las distracciones y contribuyendo a un aumento significativo tanto de la satisfacción como de la confianza en sí mismos dentro de las subdimensiones del aprendizaje. La educación basada en la simulación se ha convertido en una parte importante de la educación clínica por diversas razones, entre ellas la protección de los derechos y la privacidad de los pacientes, la falta de personal docente y de áreas de práctica suficientes, y la interrupción de la educación presencial en situaciones extraordinarias, como terremotos y pandemias^(1,13)^. Las simulaciones en pantalla, que pueden ser utilizadas tanto por individuos como por grupos, ofrecen ventajas porque el alumno puede acceder a ellas a través de una computadora, una tableta o un teléfono en cualquier momento y lugar sin necesidad de contacto presencial, y son menos costosas que los simuladores de alta fidelidad^(30,31)^.

### Limitaciones del Estudio

El estudio se limitó a los estudiantes que cursaban la asignatura de enfermería pediátrica en la Facultad de Ciencias de la Salud de la Universidad de Bal?kesir. Por lo tanto, los resultados solo pueden generalizarse a este grupo de estudio. Otra limitación del estudio puede ser que la simulación basada en pantalla no refleja completamente el entorno clínico real.

## CONCLUSIÓN

Los estudiantes de enfermería informan de que los niños experimentan estrés y baja autoeficacia y confianza en sí mismos cuando se les administran medicamentos debido a factores como la naturaleza más sensible de los niños y la dificultad de las operaciones matemáticas en la preparación de los medicamentos. Esta cuestión, tal y como se observa entre los estudiantes de enfermería, requiere una intervención específica debido a su posible impacto en la calidad de la atención de enfermería pediátrica. Por lo tanto, alcanzar altos niveles de autoeficacia, confianza en sí mismos y satisfacción es esencial para una formación eficaz en este ámbito. Por lo tanto, este estudio concluyó que los estudiantes de enfermería que recibieron formación en la administración de medicamentos a niños mediante simulación en pantalla aumentaron su autoeficacia, satisfacción y confianza en el aprendizaje en la administración de medicamentos a niños en comparación con los que recibieron formación mediante instrucciones verbales.

En contextos en los que la educación presencial no es factible, como durante desastres o pandemias, las simulaciones en pantalla ofrecen alternativas accesibles a través de dispositivos tecnológicos de uso generalizado, como teléfonos, tablets y computadoras. Esta accesibilidad contribuye positivamente a los resultados de la formación en enfermería. Los resultados de este estudio concuerdan con la bibliografía existente. Las investigaciones futuras deberían explorar las simulaciones en pantalla con estudiantes de enfermería, no solo en lo que respecta a la administración de medicamentos, sino también en diversos temas, como la comunicación con los pacientes pediátricos y sus familias, el juego terapéutico y la generalización de estos métodos. Además, se recomienda realizar estudios similares con muestras de mayor tamaño para mejorar la generalización de los resultados.

## Data Availability

Los conjuntos de datos utilizados para generar los hallazgos del estudio se pueden compartir a solicitud de los autores.
